# Supplementary material for: Differential regulation of alternative promoters emerges from unified kinetics of enhancer-promoter interaction
Source: Nat Commun. 2022 May 17;13:2714. doi: 10.1038/s41467-022-30315-6 (PMC9114328; doi:10.1038/s41467-022-30315-6)
Supplement: Supplementary file 3 — Reporting Summary [file 41467_2022_30315_MOESM3_ESM.pdf]

nature portfolio

Corresponding author(s): Heng Xu

Last updated by author(s): Apr 21, 2022

Reporting Summary

Nature Portfolio wishes to improve the reproducibility of the work that we publish. This form provides structure for consistency and transparency in reporting. For further information on Nature Portfolio policies, see our Editorial Policies and the Editorial Policy Checklist.

Statistics

For all statistical analyses, confirm that the following items are present in the figure legend, table legend, main text, or Methods section.

n/a	Confirmed	
☐	☒	The exact sample size (*n*) for each experimental group/condition, given as a discrete number and unit of measurement
☐	☒	A statement on whether measurements were taken from distinct samples or whether the same sample was measured repeatedly
☐	☒	The statistical test(s) used AND whether they are one- or two-sided<br>*Only common tests should be described solely by name; describe more complex techniques in the Methods section.*
☒	☐	A description of all covariates tested
☒	☐	A description of any assumptions or corrections, such as tests of normality and adjustment for multiple comparisons
☐	☒	A full description of the statistical parameters including central tendency (e.g. means) or other basic estimates (e.g. regression coefficient) AND variation (e.g. standard deviation) or associated estimates of uncertainty (e.g. confidence intervals)
☐	☒	For null hypothesis testing, the test statistic (e.g. *F*, *t*, *r*) with confidence intervals, effect sizes, degrees of freedom and *P* value noted<br>*Give P values as exact values whenever suitable.*
☒	☐	For Bayesian analysis, information on the choice of priors and Markov chain Monte Carlo settings
☒	☐	For hierarchical and complex designs, identification of the appropriate level for tests and full reporting of outcomes
☐	☒	Estimates of effect sizes (e.g. Cohen's *d*, Pearson's *r*), indicating how they were calculated

Our web collection on statistics for biologists contains articles on many of the points above.

Software and code

Policy information about availability of computer code

Data collection	Microscopy data was collected using Zeiss ZEN Black 2.3 SP1 and Leica LAS X 3.1.1.15751.
Data analysis	Data analysis and mathematical modeling were performed using custom MATLAB (2018a) code available at https://github.com/Dr-xu-lab/Quantify-the-transcriptional-regulation (DOI: 10.5281/zenodo.6445280). The least-squares algorithm was implemented using the “nlinfit” function in MATLAB.

For manuscripts utilizing custom algorithms or software that are central to the research but not yet described in published literature, software must be made available to editors and reviewers. We strongly encourage code deposition in a community repository (e.g. GitHub). See the Nature Portfolio guidelines for submitting code & software for further information.

Data

Policy information about availability of data

All manuscripts must include a data availability statement. This statement should provide the following information, where applicable:

- Accession codes, unique identifiers, or web links for publicly available datasets
- A description of any restrictions on data availability
- For clinical datasets or third party data, please ensure that the statement adheres to our policy

The raw image data reported in this paper are publicly accessible at a private server (http://gofile.me/4yuzx/wKna2V9pK). Source data for figures are provided with this paper.

March 2021

Field-specific reporting

Please select the one below that is the best fit for your research. If you are not sure, read the appropriate sections before making your selection.

☒ Life sciences ☐ Behavioural & social sciences ☐ Ecological, evolutionary & environmental sciences

For a reference copy of the document with all sections, see nature.com/documents/nr-reporting-summary-flat.pdf

Life sciences study design

All studies must disclose on these points even when the disclosure is negative.

Sample size	The sample size was determined based on the general standard practice in the field (Ling et al. Molecular Cell, 2019; Xu et al. Nature Methods, 2015). For each genotype and each smFISH probe set, a minimum of 7 embryos were imaged, with each containing 500-2500 imaged nuclei. For analysis with binning, the sample size of each bin is >50 promoter loci. The sample size is large enough to allow robust statistical analysis for comparing the behaviors of the two hb promoters in different genotypes.
Data exclusions	We excluded embryos in the very early or very late stages of mitotic interphase (based on nuclear morphology) because we mainly focus on steady-state transcription in this paper. Transcription in the early-stage embryo has not reached a steady state, while transcription in the late-stage embryo may have been shut down.
Replication	There were no experiments that we failed to replicate or reproduce. The numbers of biological replicates for each figure are described in figure captions.
Randomization	We controlled variability by collecting biologically independent samples in multiple batches. Embryos were allocated based on genotypes and developmental stages (nuclear cycles). Randomization was not relevant to this study as the experiments required knowledge of genotypes and developmental stages to properly compare the measured results.
Blinding	No blinding was performed since all measurements and analyses were fully automated to prevent bias.

Reporting for specific materials, systems and methods

We require information from authors about some types of materials, experimental systems and methods used in many studies. Here, indicate whether each material, system or method listed is relevant to your study. If you are not sure if a list item applies to your research, read the appropriate section before selecting a response.

Materials & experimental systems

n/a	Involved in the study	
☐	☒	Antibodies
☒	☐	Eukaryotic cell lines
☒	☐	Palaeontology and archaeology
☐	☒	Animals and other organisms
☒	☐	Human research participants
☒	☐	Clinical data
☒	☐	Dual use research of concern

Methods

n/a	Involved in the study	
☒	☐	ChIP-seq
☒	☐	Flow cytometry
☒	☐	MRI-based neuroimaging

Antibodies

Antibodies used	Rabbit anti-Bcd antibody (Santa Cruz Biotechnology, Cat#: SC-66818, Lot#: A0108) and goat anti-rabbit IgG secondary antibody conjugated with Alexa Fluor™ 488 (Invitrogen, Cat#: A11034, Lot#: 1937195).
Validation	The anti-Bcd antibody was recommended by Santa Cruz Biotechnology (https://datasheets.scbt.com/sc-66818.pdf) for detection of Bicoid of Drosophila melanogaster origin by immunofluorescence. It has been validated in Xu et al. Nature Methods (2015). The fluorophore-conjugated goat anti-rabbit IgG secondary antibody is widely used in the scientific community and was validated by the supplier (https://www.thermofisher.cn/cn/zh/antibody/product/Goat-anti-Rabbit-IgG-H-L-Highly-Cross-Adsorbed-Secondary-Antibody-Polyclonal/A-11034?adobe_mc=MCMID%7C78267828090886449162146157978355201806%7CMCAID%3D2F757D4A8515912F-40000763E5F88CEE%7CMCORGID%3D5B135A0C5370E6B40A490D44@AdobeOrg%7CTS%3D1614293705).

Animals and other organisms

Policy information about studies involving animals; ARRIVE guidelines recommended for reporting animal research

Laboratory animals	Syncytial stage embryos (without sex specification) from multiple Drosophila melanogaster strains were used in the study:<br>(1) Oregon-R (OreR) was used as the wild type.

(2) 1x bcd strain (+/CyO-bcd+; E1s) was obtained as a gift from Dr. Jun Ma (Zhejiang University).
(3) CRISPR mutant strains with P1 and P2 deletions (ΔP1C and ΔP2C) were developed in Ling et al. Mol. Cell (2019) and were obtained as gifts from Dr. Stephen Small (New York University) and Dr. Pinar Onal (Northwestern University).
(4) Strains used for enhancer deletion experiments were initially developed in Perry et al. PNAS (2011) by PhiC31-mediated recombination of genetically modified bacterial artificial chromosome (BAC) CH322-55J23 on chromosome 2 of Drosophila. The distal-enhancer-removed BAC construct and its control were integrated at the VK37 locus in the BL24872 strain (y[1] M{RFP[3xP3.PB] GFP[E.3xP3]=vas-int.Dm}ZH-2A w[*]; PBac{y[+]-attP-3B}VK00037). The proximal-enhancer-removed construct and its control were integrated at the attP40 locus in the BL25709 strain (y[1] v[1] P{y[+t7.7]=nos-phiC31\int.NLS}X; P{y[+t7.7]=CaryP}attP40). The distal-enhancer-removed BAC construct and its control were obtained as gifts from Dr. Michael Perry (University of California San Diego) and Dr. Alistair Boettiger (Stanford University). The proximal-enhancer-removed construct and its control were rebuilt.

Wild animals: This study did not involve wild animals.

Field-collected samples: This study did not involve field samples.

Ethics oversight: No ethical approval or guidance was required for the use of Drosophila melanogaster.

Note that full information on the approval of the study protocol must also be provided in the manuscript.
